# Supplementary material for: In Silico Designed Multi-Epitope Vaccine Based on the Conserved Fragments in Viral Proteins for Broad-Spectrum Protection Against Porcine Reproductive and Respiratory Syndrome Virus
Source: Vet Sci. 2025 Jun 12;12(6):577. doi: 10.3390/vetsci12060577 (PMC12197703; doi:10.3390/vetsci12060577)
Supplement: Supplementary file 1 [file vetsci-12-00577-s001.zip › Supplementry Materials File Figures S2.pdf]

Supplementary Figure

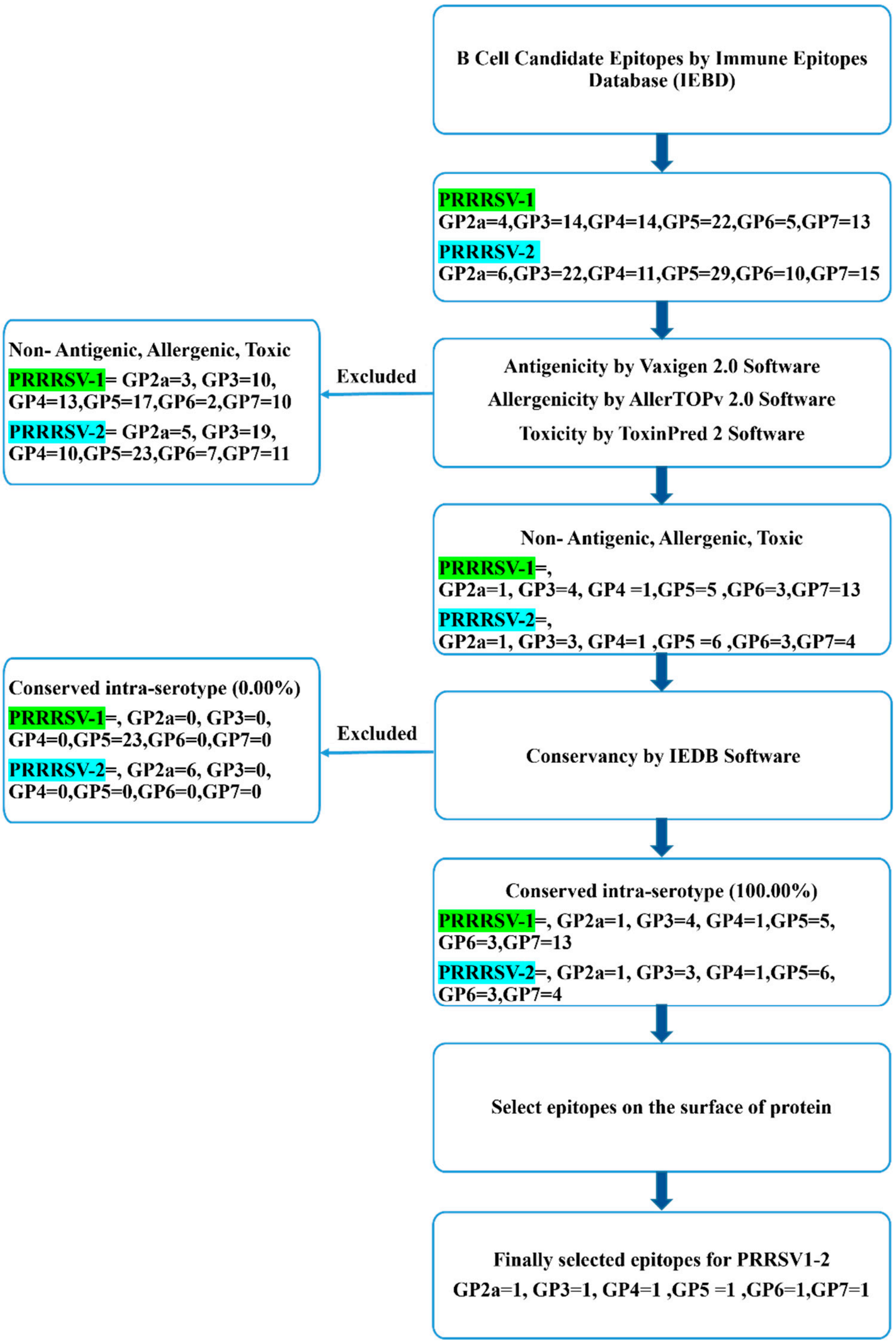

Figure S1: B Cell Epitopes and Stepwise Selection Procedure for PRRSV vaccine. This figure details the identification and properties of B cell epitopes from each protein of PRRSV, followed by a stepwise selection process that ends in determining final epitopes for vaccine design.

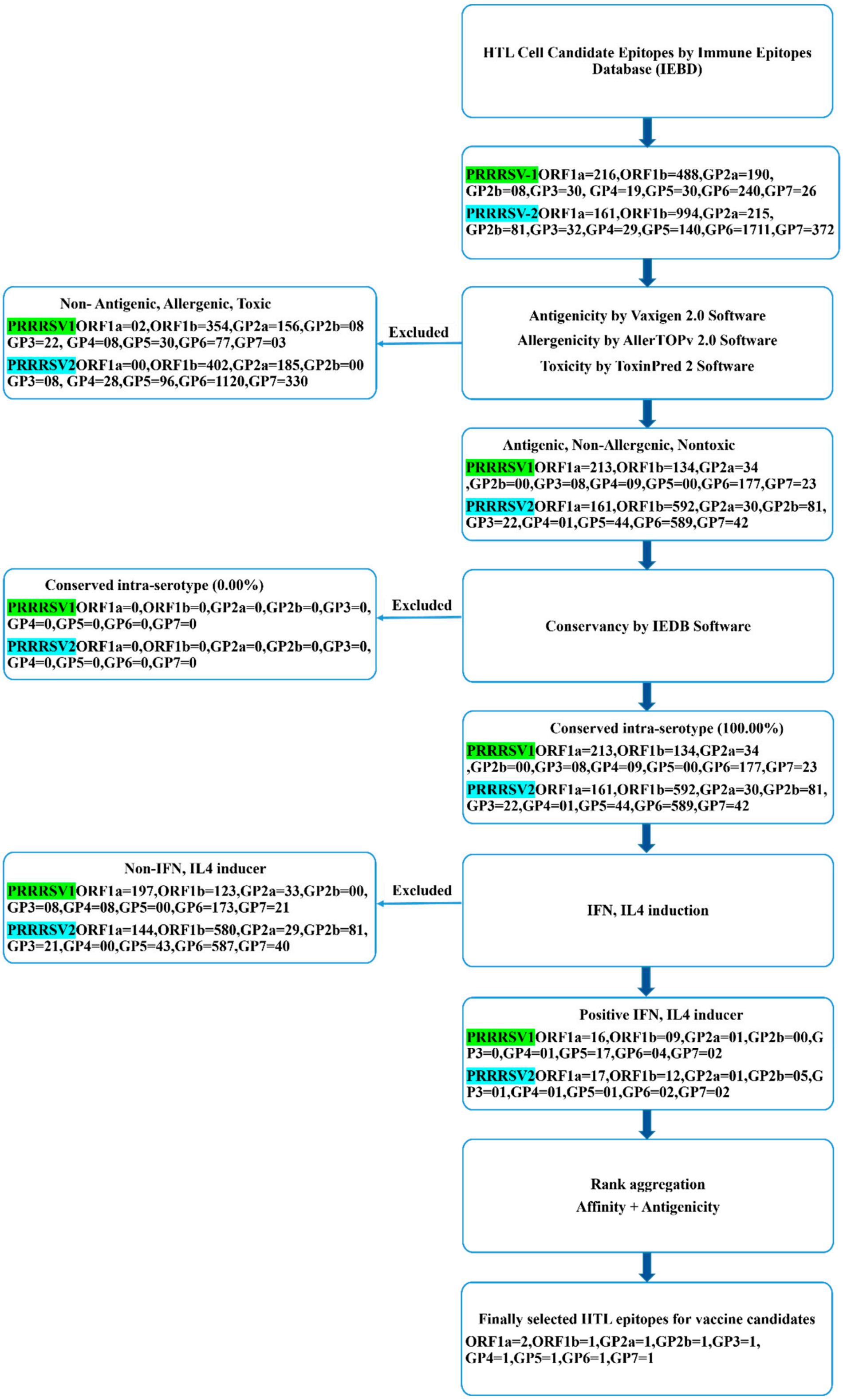
HTL Cell Candidate Epitopes by Immune Epitopes Database (IEBD)
PRRRSV-1ORF1a=216,ORF1b=488,GP2a=190, GP2b=08,GP3=30, GP4=19,GP5=30,GP6=240,GP7=26
PRRRSV-2ORF1a=161,ORF1b=994,GP2a=215, GP2b=81,GP3=32,GP4=29,GP5=140,GP6=1711,GP7=372
Antigenicity by Vaxigen 2.0 Software
Allergenicity by AllerTOPv 2.0 Software
Toxicity by ToxinPred 2 Software
Excluded
Non- Antigenic, Allergenic, Toxic
PRRRSV1ORF1a=02,ORF1b=354,GP2a=156,GP2b=08 GP3=22, GP4=08,GP5=30,GP6=77,GP7=03
PRRRSV2ORF1a=00,ORF1b=402,GP2a=185,GP2b=00 GP3=08, GP4=28,GP5=96,GP6=1120,GP7=330
Antigenic, Non-Allergenic, Nontoxic
PRRRSV1ORF1a=213,ORF1b=134,GP2a=34 ,GP2b=00,GP3=08,GP4=09,GP5=00,GP6=177,GP7=23
PRRRSV2ORF1a=161,ORF1b=592,GP2a=30,GP2b=81, GP3=22,GP4=01,GP5=44,GP6=589,GP7=42
Conservancy by IEDB Software
Excluded
Conserved intra-serotype (0.00%)
PRRRSV1ORF1a=0,ORF1b=0,GP2a=0,GP2b=0,GP3=0, GP4=0,GP5=0,GP6=0,GP7=0
PRRRSV2ORF1a=0,ORF1b=0,GP2a=0,GP2b=0,GP3=0, GP4=0,GP5=0,GP6=0,GP7=0
Conserved intra-serotype (100.00%)
PRRRSV1ORF1a=213,ORF1b=134,GP2a=34 ,GP2b=00,GP3=08,GP4=09,GP5=00,GP6=177,GP7=23
PRRRSV2ORF1a=161,ORF1b=592,GP2a=30,GP2b=81, GP3=22,GP4=01,GP5=44,GP6=589,GP7=42
IFN, IL4 induction
Excluded
Non-IFN, IL4 inducer
PRRRSV1ORF1a=197,ORF1b=123,GP2a=33,GP2b=00, GP3=08,GP4=08,GP5=00,GP6=173,GP7=21
PRRRSV2ORF1a=144,ORF1b=580,GP2a=29,GP2b=81, GP3=21,GP4=00,GP5=43,GP6=587,GP7=40
Positive IFN, IL4 inducer
PRRRSV1ORF1a=16,ORF1b=09,GP2a=01,GP2b=00,G P3=0,GP4=01,GP5=17,GP6=04,GP7=02
PRRRSV2ORF1a=17,ORF1b=12,GP2a=01,GP2b=05,G P3=01,GP4=01,GP5=01,GP6=02,GP7=02
Rank aggregation
Affinity + Antigenicity
Finally selected HTL epitopes for vaccine candidates
ORF1a=2,ORF1b=1,GP2a=1,GP2b=1,GP3=1, GP4=1,GP5=1,GP6=1,GP7=1

Figure S2: HTL (MHC2 Cell Epitopes and Stepwise Selection Procedure for PRRSV vaccine. This figure details the identification and properties of HTL cell epitopes from each protein of PRRSV, followed by a stepwise selection process that ends in determining final epitopes for vaccine design.

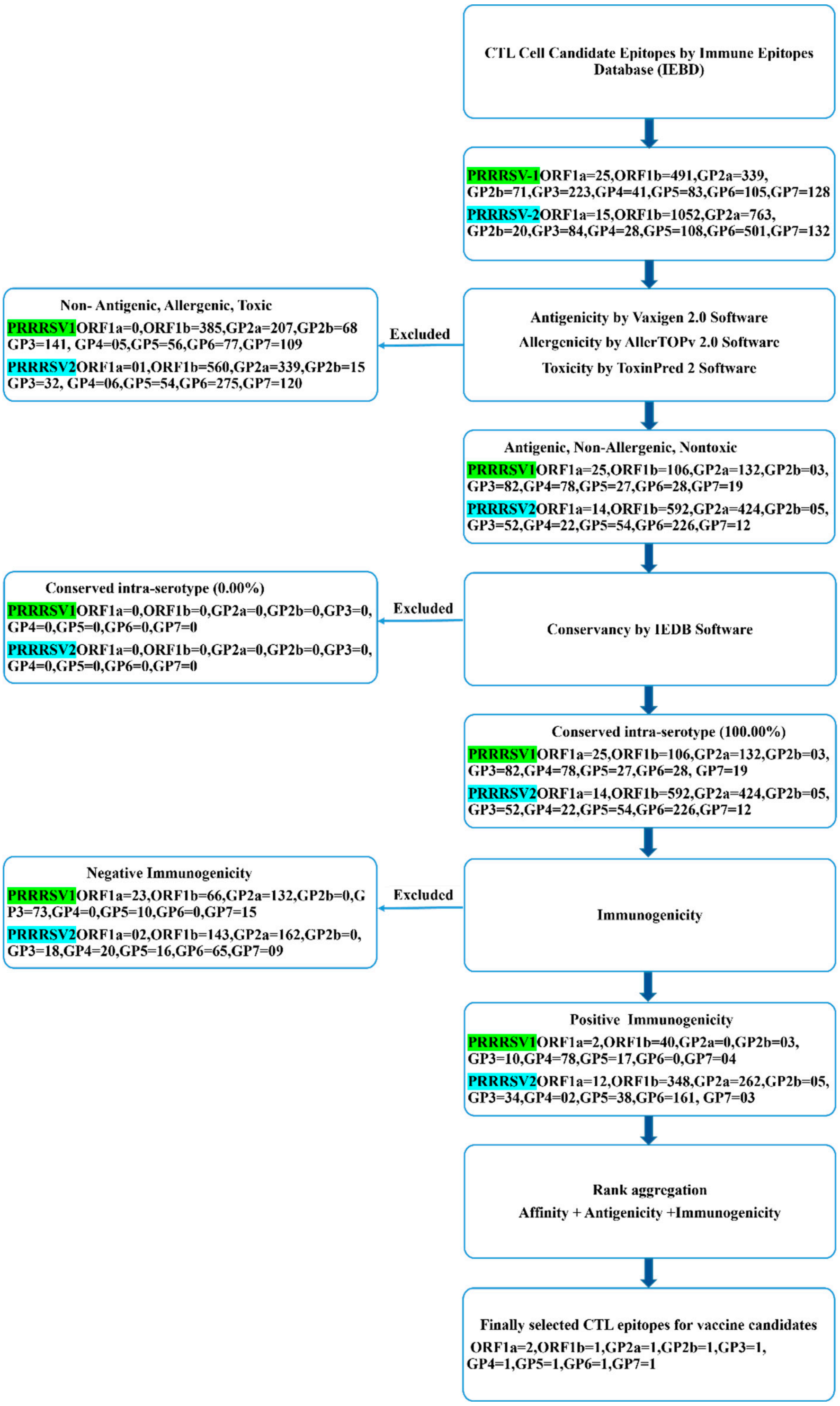

Figure S3: CTL Cell (MHC1) Epitopes and Stepwise Selection Procedure for PRRSV vaccine design. This figure details the identification and properties of CTL cell epitopes from each protein of PRRSV, followed by a stepwise selection process that ends in determining final epitopes for vaccine design.

A PRRSV-V-1 531 Amino acid

MSDINKLAETLVNLKIVEVNDLAKILKEKYGLDPSANLAIPSLPKAEILDKSKEKTSFDLILKGAGSAKLTVVK
RIKDLIGLGLKESKDLVDNVPKHLKKGLSKEEAESLKKQLEEVGAEVELKEAAAKAPRYSVRALPKKLVRGN
FSFKKQHTQQHHLVIDHIRKKLNGTDWLDKRFDWKKRKPGLTSVNGTLVKKHFPLAAEDGPGPGGVAPAVRI
AERYRGRGPGPGKPIAYANLDEKKISAGPGPGCLGDFKQLHPVGFDSGPGPGLSFASDWFAPRYSVRGPGPGN
WFHLEWLRPFFSSWGPGPGACVNFTDYVAHVTQHGPGPGWRYSCTRYTNFLLDTGPGPGLAFSITYTPIIYAL
KGPGPGPHFPLATEDDVRHHFGPGPGVFCIRLVCSAIHRSRAAYAALTGRTLAAYHQKPIAYANLAAYTRARHA
IFAAYSVRALPFTLAAYIFLAILFGFAAYYAWLAFLSFAAYSACVNFTDYAAYTRYTNFLLAAYFSITYTPIIAAYF
PLATEDDVRHHF

B PRRSV-V-2 448 Amino acid

GIINTLQKYYCRVRGGRCAVLSCLPKEEQIGKCSTRGRKCCRRKKEAAAKAPRYSVRALPKKLVRGNFSF
KKQHTQQHHLVIDHIRKKLNGTDWLDKRFDWKKRKPGLTSVNGTLVKKHFPLAAEDGPGPGGVAPAVRI
AERYRGRGPGPGKPIAYANLDEKKISAGPGPGCLGDFKQLHPVGFDSGPGPGLSFASDWFAPRYSVRGPGP
GNWFHLEWLRPFFSSWGPGPGACVNFTDYVAHVTQHGPGPGWRYSCTRYTNFLLDTGPGPGLAFSITYT
PIIYALKGPGPGPHFPLATEDDVRHHFGPGPGVFCIRLVCSAIHRSRAAYAALTGRTLAAYHQKPIAYANLA
AYTRARHAIFAAYSVRALPFTLAAYIFLAILFGFAAYYAWLAFLSFAAYSACVNFTDYAAYTRYTNFLLAAY
FSITYTPIIAAYFPLATEDDVRHHF

C PRRSV-V-3 551 Amino acid

MAENPNIDDLPAPLLAALGAADLALATVNDLIANLRERAEETRAETRTRVEERRARLTKFQEDLPEQFIELRD
KFTTEELRKAAEGYLEAATNRYNELVERGEAALQRLRSQTAFEDASARAEGYVDQAVELTQEALGTVASQT
RAVGEAAAKAPRYSVRALPKKLVRGNFSFKKQHTQQHHLVIDHIRKKLNGTDWLDKRFDWKKRKPGLTSV
NGTLVKKHFPLAAEDGPGPGGVAPAVRIAERYRGRGPGPGKPIAYANLDEKKISAGPGPGCLGDFKQLHPVGF
DSGPGPGLSFASDWFAPRYSVRGPGPGNWFHLEWLRPFFSSWGPGPGACVNFTDYVAHVTQHGPGPGWRYS
CTRYTNFLLDTGPGPGLAFSITYTPIIYALKGPGPGPHFPLATEDDVRHHFGPGPGVFCIRLVCSAIHRSRAAYA
ALTGRTLAAYHQKPIAYANLAAYTRARHAIFAAYSVRALPFTLAAYIFLAILFGFAAYYAWLAFLSFAAYSACV
NFTDYAAYTRYTNFLLAAYFSITYTPIIAAYFPLATEDDVRHHF

Figure S4: Peptide Sequences of designed vaccine model of PRRSV virus. **(A)**PRRSV-V-1, 531 aa Sequences, **(B)**PRRSV-V-2, 448 aa Sequences, **(B)**PRRSV-V-3, 551 aa Sequences

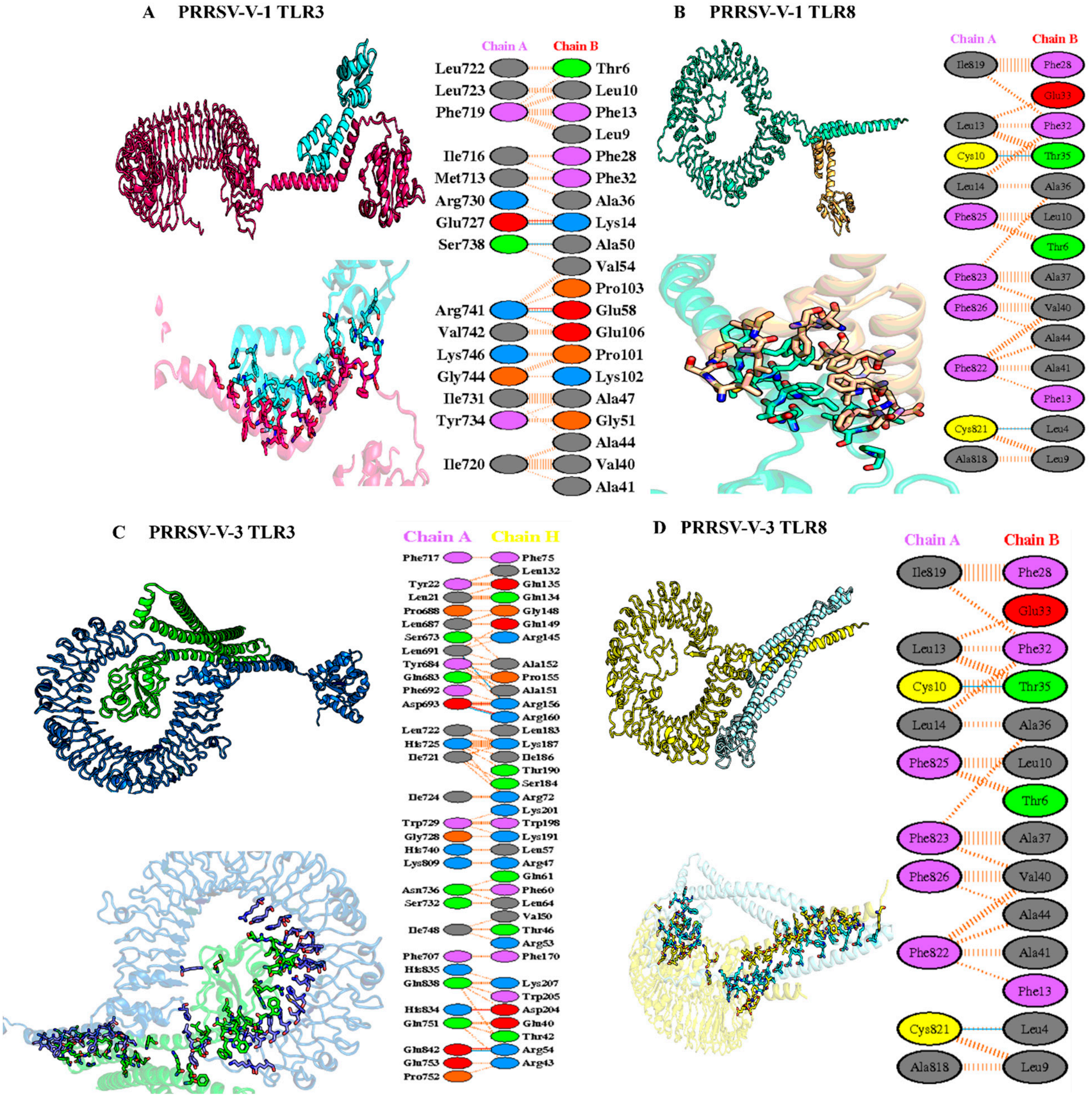

Figure S5: Analysis of docking modeled vaccine with TLL3 and TLR8 (A and B) PRRSV-V-1 interaction with TLR3 and TLR8, **(C and D)** PRRSV-V-3 interaction with TLR3 and TLR8.

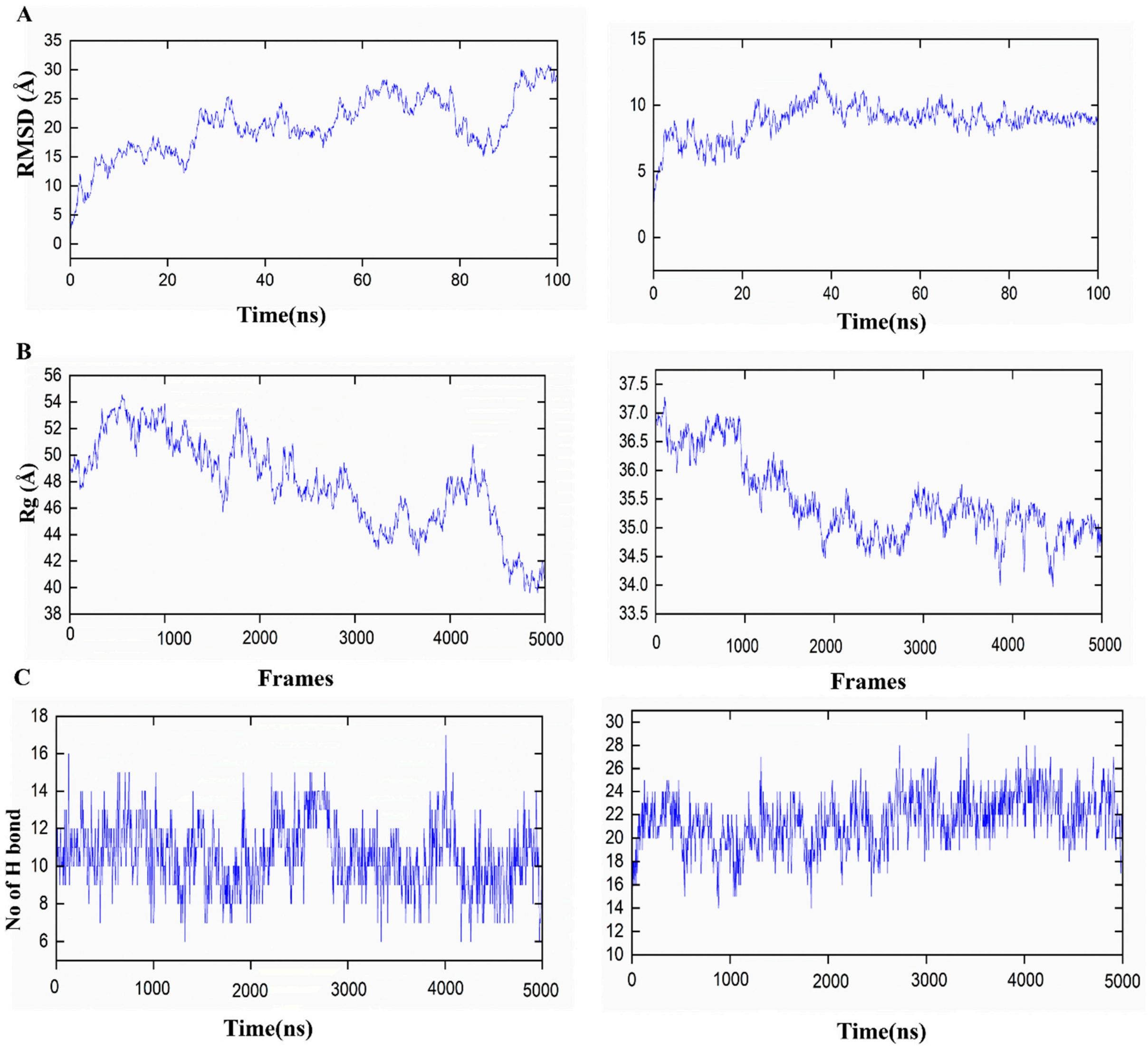

Figure S6: Results of Molecular Dynamic Simulation(A) Root means square deviation (RMSD): shows the RMSD for the complexes of PRRSV-V-1 with TLR3 and TLR8. **(B)**Radius of Gyration (Rg): Rg is the compactness of PRRSV-V-1 with TLR3 and TLR8. **(D)**Hydrogen Bonds: the count of intra-molecule hydrogen bonds formed by PRRSV-V-1 with TLR3 and TLR8.

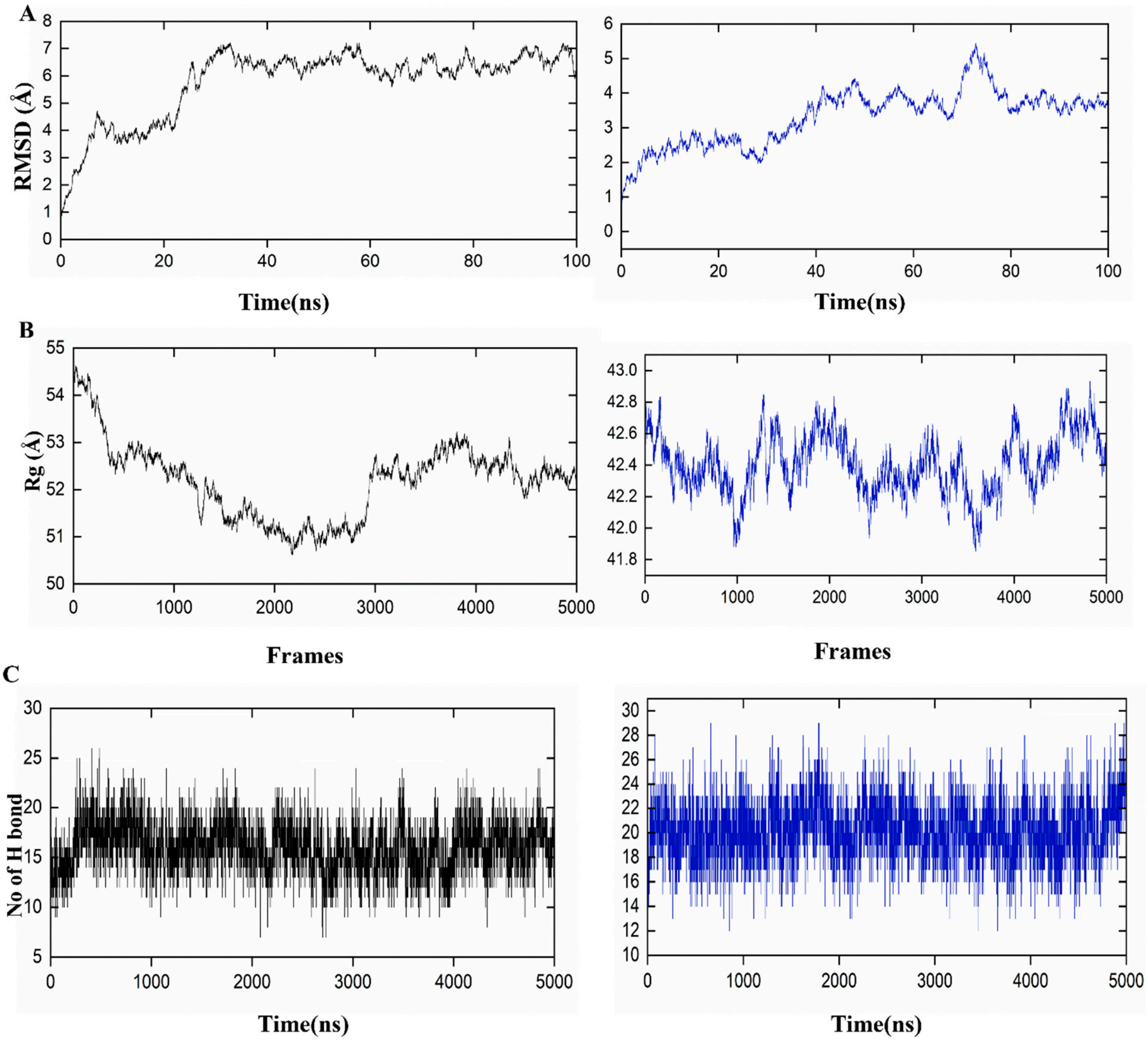

Figure S7: Results of Molecular Dynamic Simulation(A) Root means square deviation (RMSD): shows the RMSD for the complexes of PRRSV-V-3 with TLR3 and TLR8 **(B)**Radius of Gyration (Rg): Rg the compactness of PRRSV-V-3 with TLR3 and TLR8, **(C)**Hydrogen Bonds: the count of intra-molecule hydrogen bonds formed of PRRSV-V-3 with TLR3 and TLR8.

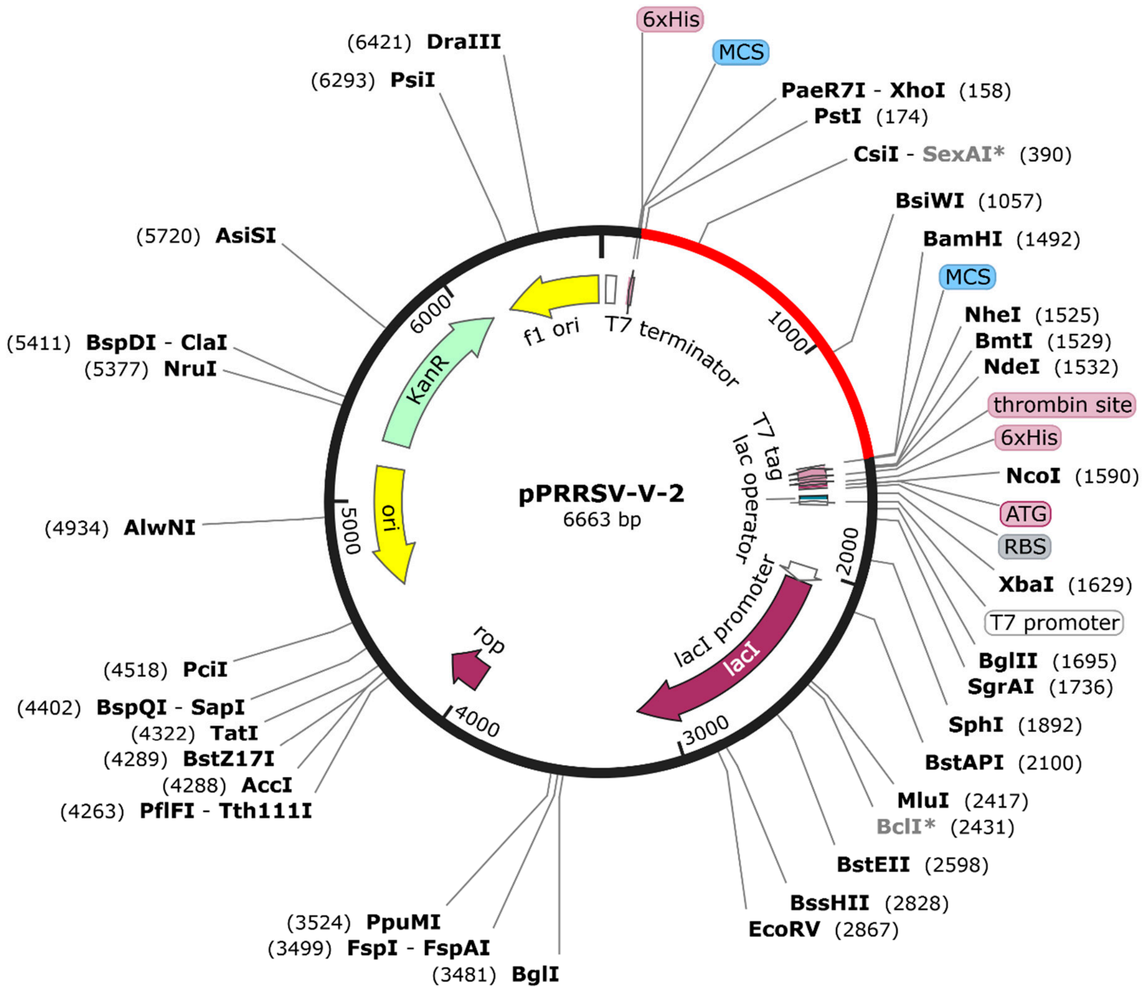

Figure S8: pPRRSV-V-2 codon optimization and plasmid vector construction. The final construct was adapted for use in the pET-28a (+) to form the recombinant plasma sequence of protein expression. The red sequence (gene of interest) is the PRRSV codon sequence optimized for the final designed vaccine. The cloning was done in SnapGene 7.0.2.
